# Disrupted longitudinal restoration of brain connectivity during weight normalization in severe anorexia nervosa

**DOI:** 10.1038/s41398-023-02428-z

**Published:** 2023-04-28

**Authors:** Lisa-Katrin Kaufmann, Jürgen Hänggi, Lutz Jäncke, Volker Baur, Marco Piccirelli, Spyros Kollias, Ulrich Schnyder, Chantal Martin-Soelch, Gabriella Milos

**Affiliations:** 1 Department of Consultation-Liaison Psychiatry and Psychosomatics, University Hospital Zurich, University of Zurich, Zurich, Switzerland; 2 Division of Neuropsychology, Department of Psychology, University of Zurich, Zurich, Switzerland; 3 Translational Research Center, University Hospital of Psychiatry, University of Bern, Bern, Switzerland; 4 University Research Priority Program (URPP) “Dynamic of Healthy Aging”, University of Zurich, Zurich, Switzerland; 5 Department of Neuroradiology, University Hospital Zurich, Zurich, Switzerland; 6 University of Zurich, Zurich, Switzerland; 7 Unit of Clinical and Health Psychology, Department of Psychology, University of Fribourg, Fribourg, Switzerland

**Keywords:** Psychiatric disorders, Neuroscience

## Abstract

Altered intrinsic brain connectivity of patients with anorexia nervosa has been observed in the acute phase of the disorder, but it remains unclear to what extent these alterations recover during weight normalization. In this study, we used functional imaging data from three time points to probe longitudinal changes in intrinsic connectivity patterns in patients with severe anorexia nervosa (BMI ≤ 15.5 kg/m^2^) over the course of weight normalization. At three distinct stages of inpatient treatment, we examined resting-state functional connectivity in 27 women with severe anorexia nervosa and 40 closely matched healthy controls. Using network-based statistics and graph-theoretic measures, we examined differences in global network strength, subnetworks with altered intrinsic connectivity, and global network topology. Patients with severe anorexia nervosa showed weakened intrinsic connectivity and altered network topology which did not recover during treatment. The persistent disruption of brain networks suggests sustained alterations of information processing in weight-recovered severe anorexia nervosa.

## Introduction

Anorexia nervosa (AN) is a severe and enduring eating disorder, with an estimated recovery rate of only 46% [1, 2]. Treatment for severe anorexia nervosa typically consists of intensive specialized inpatient therapy, but long-term treatment outcomes are often not satisfactory and the illness is extremely persistent [2].

Investigations into the neural basis underlying AN using resting-state approaches that examine functional relationships throughout the brain have provided evidence of impaired intrinsic connectivity [3–5]. Resting-state approaches provide information on task-independent intrinsic connectivity and are a powerful tool to explore alterations in brain circuitry [6] with high predictive value for behavioral measures [7]. During the state of acute underweight, studies have provided evidence for altered intrinsic connectivity in AN [3, 5]. However, closer examination reveals that the results are inconsistent with respect to direction and location. For example, both underconnectivity [8–11] and overconnectivity [12–14] of networks involving the insula are reported in acute AN. The inferior frontal gyrus has similarly been described as both underconnected [14, 15] and overconnected [16]. Even region-specific seed-based approaches focusing on the nucleus accumbens, for example, report both over- [17] and underconnectivity [18]. While data-driven methods tend to show underconnectivity in patients with AN [8–10], there is great heterogeneity in terms of the regions and networks examined, such that there is currently no consensus on alterations of intrinsic connectivity in the acute state of AN.

Another open question is how these widespread alterations develop over the course of weight normalization. Longitudinal studies investigating restoration processes during weight normalization are scarce [3]. To date, only four longitudinal studies have examined weight-related changes of connectivity in adolescents and young adults. Following weight normalization, they report mixed findings of normalization of nucleus accumbens overconnectivity [17, 19], full restoration of underconnectivity [20], as well as persisting underconnectivity between the salience and executive control networks [21]. In weight-recovered adults, there are mixed reports of no residual alterations [20], underconnectivity in visual and auditory resting-state networks [22, 23], and overconnectivity in the default mode network [16]. These incongruent findings could be influenced not only by disparate methodology but also by the age range of patients. Results from whole-brain approaches are mostly based on adolescent samples at an early stage of disease [24] or on mixed samples of adolescents and adults [8, 25]. Given that brain development during adolescence follows a nonlinear course [26, 27], it is particularly difficult to control for developmental influences. Critically, no longitudinal functional connectivity study to date has examined whether intrinsic connectivity alterations recover following weight normalization in adult patients with severe AN.

Complementing large-scale connectivity analyses, the network architecture of the brain can be described in terms of two key properties: the functional segregation of specialized brain regions and the concurrent integration of information [28]. Graph-theoretical measures describe the topology of brain organization by quantifying reductions in short-range connections (segregation) and increases in long-range connections (integration) [29, 30], which represents a trade-off between wiring costs and the efficiency of information transfer [30]. At the structural level, AN has recently been associated with a decrease in network segregation [31]. Only three studies have examined network topology in the functional domain, reporting mixed findings of decreased network integration (i.e., greater characteristic path length and lower betweenness centrality) in adolescents and young adults [9, 32] and decreased network segregation (i.e., lower global clustering) in adult patients [33]. The influence of the age of the patients is yet unclear. A first study on recovered anorexia patients suggests that aberrant network topology may persist after weight normalization [34]. Importantly, none of the previous studies employed a longitudinal approach and have yet to be replicated in independent samples.

**Fig. 1:**
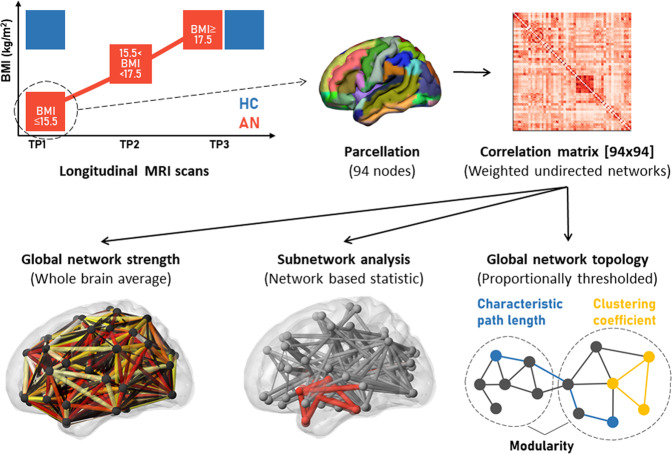
Schematic illustration of study procedure and methods. Brain images of patients with anorexia nervosa (AN) were acquired at three time points (TP1, TP2, TP3) over the course of weight normalization. Healthy controls (HC) were scanned twice (TP1, TP3). Functional MRI data were processed and parcellated with an adapted version of the automated anatomical labelling atlas with 94 nodes [44]. Intrinsic connectivity was calculated as Pearson correlation between the mean time series of any pair of two nodes [94×94], standardized with Fisher’s r-to-z transformation. Global network strength was computed as the whole brain average of all positive connections per person. Subnetworks of altered connections were analyzed using the network-based statistic tool [47]. Global network topology was captured using characteristic path length (shortest average path length) as a measure of integration, and clustering coefficient (ratio between closed triplets relative to all triplets) and global modularity (ratio between within cluster connections to all connections) as a measure of segregation [29].

Here, we investigated intrinsic connectivity in a longitudinal design with three time points to elucidate network alterations and recovery processes during treatment in women with severe AN. We examined global network strength, subnetworks with differences in neural synchrony, and global network topology in patients with severe AN and closely matched healthy controls (HC). Intrinsic connectivity was assessed in women with AN at (TP1) the beginning of treatment with severe underweight, (TP2) after initial weight gain, and at (TP3) the end of treatment with an approximately healthy body mass index (BMI). We tested the hypothesis that network underconnectivity and altered topology within the patient group restore with weight normalization over the course of inpatient treatment (Fig. 1).

## Methods and materials

### Participants

Twenty-eight women with severe AN (aged 18–32) and 40 HC (aged 18–30) participated in the study. One patient had to be excluded due to MRI artefacts, leaving the data of 27 women with AN (24 restrictive type, 3 binge-purge type) for analysis. The two groups were matched for sex, handedness, age, intelligence, and years of education at the group level (Table 1). This sample partially overlaps with previous studies of our group [35, 36]. Inclusion criteria for patients comprised severe AN (BMI ≤ 15.5 kg/m^2^) and an illness duration >1 year. Patients were enrolled in an eating disorder-specific inpatient program with a target BMI ≥ 18.5 kg/m^2^, that included individual and group psychotherapy, somatic controls, structured dietary increase, nutritional counselling, body-perception and art therapy, physiotherapy, and cooking groups. Sixteen patients were receiving psychotropic medication when they entered the study and continued to take them unchanged (see Supplemental Methods). Healthy controls were required to have no history of mental illness, no first- or second-degree relatives with a lifetime diagnosis of an eating disorder, and no medications, including hormonal contraceptives. All participants provided written informed consent prior to participation. The study protocol complied with the Declaration of Helsinki and was approved by the local ethics review board.

**Table 1:** Group characteristics.

	Group and time point							
	AN1 *n* = 27	AN2 *n* = 27	AN3 *n* = 27	HC1 *n* = 40	HC3 *n* = 40	AN1 v. HC1	AN3 v. HC3	AN1 v. AN3
	Mean *(SD)*	Mean *(SD)*	Mean *(SD)*	Mean *(SD)*	Mean *(SD)*	*p*	*p*	*p*
Age	22.04 (3.97)			23.25 (3.27)		0.195		
Education	13.31^a^ (2.46)			14.53^d^ (4.14)		0.146		
BMI	14.18 (1.03)	16.55 (0.71)	18.46 (0.47)	20.72 (1.67)	20.45 (1.51)	<0.001	<0.001	<0.001
BMI increase per week		0.24 (0.06)	0.20 (0.08)					
Weeks after previous TP		10.42 (3.31)	12.69 (14.13)		31.24 (18.45)			
EDE-Q total	3.25^a^ (1.31)	2.01^a^ (1.13)	1.71^c^ (1.06)	0.61^d^ (0.52)	0.48^c^ (0.48)	<0.001	<0.001	<0.001
EDE-Q eating concern	3.19^a^ (1.55)	1.68^a^ (1.12)	1.41^c^ (1.01)	0.28^d^ (0.52)	0.23^c^ (0.37)	<0.001	<0.001	<0.001
EDE-Q restraint	3.30^a^ (1.72)	1.19^a^ (1.08)	1.03^c^ (0.81)	0.46^d^ (0.56)	0.27^c^ (0.34)	<0.001	<0.001	<0.001
EDE-Q shape concern	3.63^a^ (1.30)	3.10^a^ (1.59)	2.57^c^ (1.64)	0.92^d^ (0.81)	0.82^c^ (0.71)	<0.001	<0.001	0.008
EDE-Q weight concern	2.87^a^ (1.21)	2.07^a^ (1.25)	1.84^c^ (1.44)	0.76^d^ (0.72)	0.62^c^ (0.73)	<0.001	0.001	0.005
BDI	23.62^a^ (9.66)	17.96^b^ (10.14)	12.26^c^ (10.09)	3.74^d^ (4.36)	2.67^c^ (2.69)	<0.001	<0.001	<0.001
WMT	121.63 (17.71)			127.54^e^ (15.99)		0.180		
WST	106.35^a^ (8.65)			105.26^e^ (10.77)		0.663		

*Notes*. Clinical and psychometric measures were compared using two-tailed Welch’s t-tests. Age and education are reported in years. *P*-values are adjusted for multiple comparisons using the Holm−Bonferroni procedure (Holm, 1979). *BMI* body mass index, *BDI* Beck Depression Inventory (maximum score: 63, clinically meaningful cut-off: 18), *EDE-Q* Eating Disorder Examination Questionnaire score (maximum total score: 6), *TP* Time point, *WMT* Viennese Matrices Test, *WST* Multiple Choice Vocabulary Test.

Missing data: ^a^*n* = 1, ^b^*n* = 3, ^c^*n* = 4, ^d^*n* = 2, ^e^*n* = 5.

### Procedure

Patients’ brain images were acquired at the beginning of treatment (TP1, BMI ≤ 15.5 kg/m^2^), after an initial weight gain of approximately 2 BMI points (TP2, 15.5 < BMI < 17.5 kg/m^2^), and at the end of treatment (TP3, BMI of ≥ 17.5 kg/m^2^). Healthy controls underwent the same procedure at two time points (corresponding to TP1 and TP3, see Fig. 1). As intrinsic connectivity can be influenced by hormonal alterations across the menstrual cycle [37, 38] and the large majority of patients with AN were amenorrhoeic (85%, details are provided in Supplemental Methods), HC were scanned in the follicular phase within the first 10 days of their cycle. Both groups underwent the same standardized intake procedure (see Supplemental Methods), and scanning for all participants took place between 3:00 and 4:30 pm. Eating disorder psychopathology was assessed at all measurement time points with the validated German version of the Eating Disorder Examination Questionnaire (EDE-Q) [39] and symptoms of depression were quantified with the Beck Depression Inventory (BDI) [40]. Intelligence was estimated using the Viennese Matrices Test (WMT) for fluid intelligence [41] and the Vocabulary test (WST) for verbal intelligence [42].

### MRI data acquisition and preprocessing

T1-weighted structural images and resting-state functional images were acquired using a 3.0 Tesla whole-body magnetic resonance imaging (MRI) system (Ingenia, Philips Healthcare, Best, The Netherlands) equipped with a 32-channels receive phased array head coil (see Supplement for further acquisition details). Images of all subjects were inspected by a trained neuroradiologist for any relevant pathology. Functional MRI data were processed with the DPARSFA toolbox (version 4.5, RRID:SCR_002372) [43]. An adapted version of the automated anatomical labelling (AAL) atlas with 94 regions was used to define the network nodes [44], including the nucleus accumbens and the anterior and posterior parts of the insular cortex (Table S1). Intrinsic connectivity strength was calculated as Pearson correlation between the mean time series of any pair of two nodes, standardized with Fisher’s *r*-to-*z* transformation (see Supplemental Methods for further preprocessing details). In addition, regional homogeneity images were calculated as Kendall’s coefficient of concordance between the time series of a given voxel with those of its 26 neighboring voxels [45]. To test whether potential network disruptions would be explained by local reductions in neuronal synchrony, mean regional homogeneity per node was extracted for group comparisons (see Supplement).

### Global strength

Global strength was computed as the mean strength of all connections per person, excluding negative connections and the matrix diagonal. We compared global strength across time using a mixed ANOVA (group × time), between groups at TP1 and TP3, and within the AN group across time (TP1, TP2, TP3) using two-tailed Welch’s *t*-tests. Standardized mean differences are reported as Hedges’ *g* [46].

### Subnetwork analyses

The network-based statistic (NBS) toolbox (RRID:SCR_002454) [47] was used in a MATLAB environment (R2019b, RRID:SCR_001622) to test for subnetworks of altered connections, while controlling the family-wise error (FWE) [47]. To assess group differences, functional connectomes were compared between groups at TP1 and TP3. To examine changes over time, we compared both groups across time (group × time), and tested for changes within the AN group (between TP1 and TP2, and between TP2 and TP3). Group comparisons were performed for both contrasts (AN < HC and AN > HC) and all analyses were performed with the total number of connections as a measure of component size, controlled for multiple comparisons using 5,000 permutations at *p* ≤ 0.05. To minimize the rate at which the null hypothesis for individual connections was falsely rejected, the primary sensitivity threshold for the between-group analysis at TP1 and TP3 was set to *t* = 3.0 (corresponding to Cohen’s *d* ≥ 0.7). A more sensitive primary threshold of *t* = 1.0 (*d* ≥ 0.2) and *F* = 1.5 (*d* ≥ 0.2) was chosen for the longitudinal analyses to detect changes over time. To assess the robustness of results with alternative thresholds, our findings were repeated with different primary thresholds (see Supplemental Results). Additionally, to further examine recovery processes during treatment within the AN group, the mean connectivity of the network identified at TP1 was extracted and analyzed for significant changes over time, using Welch’s *t*-tests for dependent samples.

### Global network topology

To calculate the graph metrics, individual connectivity matrices were thresholded at proportional density thresholds between 0.05 and 0.45 at 0.01 intervals. Importantly, we preserved the connection weights as weighted networks have been found to offer better reliability by capturing more comprehensive topological information [48]. Negative correlations and the matrix diagonal were excluded from the analyses. Using the Network Toolbox for R [49], we calculated characteristic path length (global average shortest path length, i.e. geodesic distance) as a measure of integration, global clustering coefficient as a measure of segregation (with weights converted to values between 0 and 1), and global modularity as a measure of compartmentalization of communities [29] (Fig. 1). Integration refers to the connectedness of nodes within a network, highly integrated networks have shorter average path length. Segregation refers to the extent to which networks show densely interconnected groups of nodes, higher clustering coefficient values meaning higher segregation. Modularity is related to the ratio between within cluster connections to all connections and quantifies the segregation into functionally meaningful clusters. Higher modularity indicates segregation between clusters [29, 30].

Group comparisons were performed using two-tailed Welch’s *t*-tests and cluster-based permutation testing with 10,000 permutations in R (version 4.2.2, RRID:SCR_001905) [50] to control the FWE rate. Cluster-based permutation has the advantage of greater sensitivity and robustness compared to the conventional area-under-the-curve approach [51], where only the integral over the range of thresholds is considered for group comparison. The probability for clustered differences between groups was estimated under the null distribution, preserving the dependency across thresholds within a topological measure by maintaining the shuffling of group labels within each permutation. Clusters of group differences were defined by *p* < 0.05. The empirical cluster size *k* was compared with the cluster sizes from the permutation-based null distribution, calculating the *p*-value as the proportion of clusters ≥ *k* under the null distribution (α = 0.05).

### Correlation analyses

To test for covariation between measures of functional connectivity and clinical (BMI, age, age of onset, illness duration) and psychometric eating disorder parameters (EDE-Q, BDI) within the patient group, Pearson correlations were calculated using R (version 4.2.2, RRID:SCR_001905) [50]. For the analyses of global network topology, mean values were calculated across the range of proportional thresholds for each graph metric. The significance level for the correlation analyses was set to α = 0.05, adjusted for multiple comparisons using the Holm−Bonferroni procedure [52].

## Results

### Clinical and psychometric measures

Patients with severe AN were on average 16.30 ± 2.84 years at illness onset and had a mean illness duration of 5.88 ± 4.18 years. Relative to their age-, education-, and intelligence-matched peers in the control group, the AN group showed elevated scores for eating disorder-related cognition (EDE-Q) and depression (BDI), and lower BMI at baseline. These differences decreased over the course of treatment but remained significant at the end of treatment compared with matched HC (Table 1).

### Global strength

**Fig. 2:**
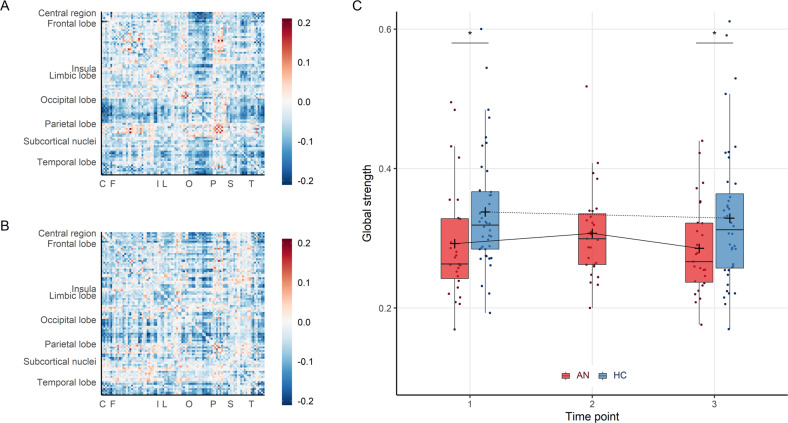
Connectivity strength in patients with severe AN compared to HC. **A**, **B** Functional connectivity between 94 regions of interest, difference between groups (AN minus HC) at TP1 **A** and TP3 **B** show global underconnectivity at both time points. C = central region, F = frontal lobe, I = insula, L = limbic lobe, O = occipital lobe, P = parietal lobe, S = subcortical nuclei, T = temporal lobe. **C** Boxplots of global strength per group and time point. Patients with AN (red) show persistent underconnectivity compared to healthy controls (blue). The cross signifies the mean, the horizontal mark signifies the median, edges of the box represent 25th and 75th percentiles, and the whiskers extend to 1.5 interquartile ranges. *p < 0.05.

The first aim was to examine whether intrinsic connectivity of patients with AN in the phase of severe underweight differed from HC. Results of the group comparison at TP1 showed lower global connectivity strength of patients (AN1: mean = 0.29 ± 0.08) compared with HC (HC1: mean = 0.33 ± 0.10, *t*(57.02) = -2.166, *p* = 0.035, *g* = -0.532) (Fig. 2A+C).

Over the course of treatment, there was no evidence for a group × time interaction (*F*(1, 65) = 0.011, *p* = 0.918) or changes in global strength within the group of AN patients between TP1 (AN1: mean = 0.29 ± 0.08) and TP2 (AN2: mean = 0.31 ± 0.07, *t*(26) = 1.019, *p* = 0.317, *g* = 0.19) and between TP2 and TP3 (AN3: mean = 0.29 ± 0.07, *t*(26) = −1.457, *p* = 0.157, *g* = −0.32). Compared with HC, global strength remained significantly lower in patients with AN at TP3 (HC3: mean = 0.33 ± 0.10, *t*(64.97) = −2.107, *p* = 0.039, *g* = −0.498) (Fig. 2B+C). There was no evidence for associations between global strength and eating disorder parameters (clinical and psychometric) within the patient group (see Supplemental Results).

### Subnetwork analyses

Consistent with the results of weakened global connectivity strength, NBS analyses yielded a subnetwork of underconnectivity in patients with AN compared with HC at TP1 (*p* < 0.004, FWE-corrected). The subnetwork comprised 61 nodes and 125 connections, spanning nearly the whole brain (Fig. 2A, Table S2). Regional homogeneity of these nodes did not differ between groups (Table S4). There was no evidence for overconnectivity in patients with severe AN.

**Fig. 3:**
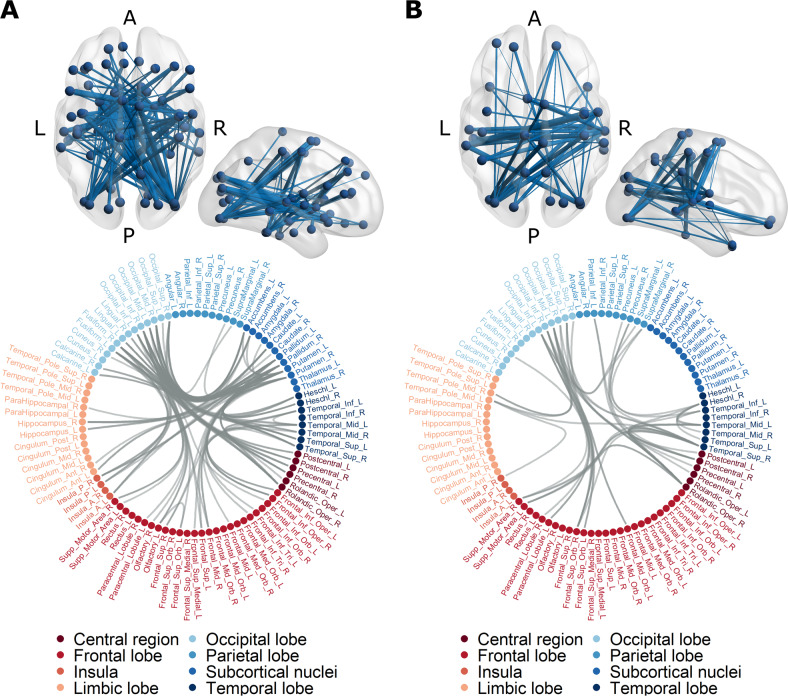
Subnetworks of underconnectivity in patients with severe AN compared to HC. Network nodes were defined using an adapted version of the automated anatomical labelling atlas with 94 regions [44]. **A** At TP1, patients with AN showed decreased functional connectivity in a large subnetwork, encompassing subcortical, limbic, insular, frontal, central, occipital, and temporal regions (*p*_FWE_ < 0.004, nodes 61, connections 125). **B** At TP3, a subnetwork of persistent underconnectivity in patients with AN emerged (*p*_FWE_ = 0.0308; nodes 33, connections 48).

With weight normalization, follow-up analysis of the subnetwork identified at TP1 revealed an increase of mean connectivity within the subnetwork in the patient group between TP1 and TP2 (mean difference = 0.05 ± 0.11, *t*(26) = 2.647, *p* = 0.014, *g* = 0.62), indicating that the weakened network slightly recovered with weight normalization. Between TP2 and TP3, mean connectivity of the subnetwork remained largely unchanged (mean difference = −0.02 ± 0.10, *t*(26) = −0.863, *p* = 0.396, *g* = −0.20). Whole-brain analyses within the AN group, between TP1 and TP2, and between TP2 and TP3, yielded no evidence of additional subnetworks over the course of weight normalization. Similarly, comparison of both groups in a whole-brain ANOVA (group × time) yielded no evidence of network changes over the course of treatment. A whole-brain comparison of the groups at TP3 revealed a subnetwork of persistent underconnectivity (*p* < 0.004, FWE-corrected). This subnetwork was less extensive compared with TP1, comprising 33 nodes and 48 connections (Fig. 3B, Table S3). There was no evidence for associations between subnetwork connectivity and eating disorder parameters (clinical and psychometric) within the patient group (see Supplemental Results).

### Global network topology

**Fig. 4:**
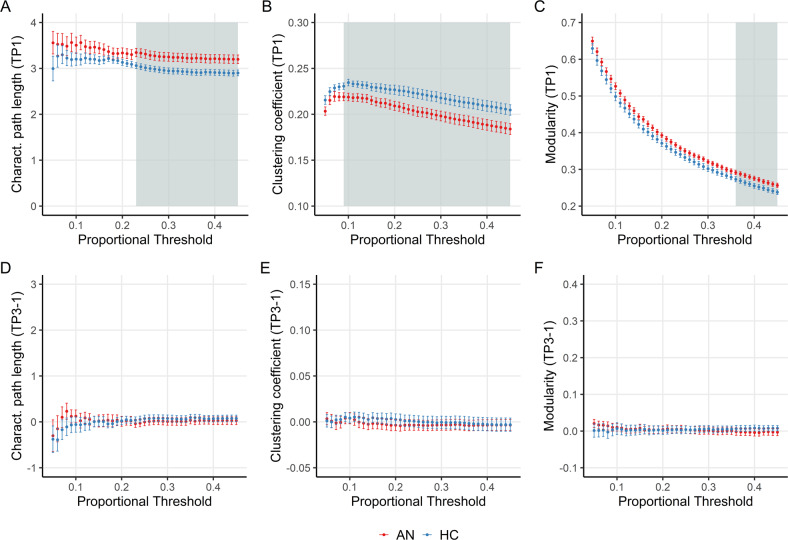
Global network topology compared between patients with AN (red) and healthy controls (blue). Proportional thresholds ranged from 0.05 to 0.45 at 0.01 intervals. **A**–**C** At TP1, cluster-based permutation testing revealed statistically significant group differences (gray-shaded areas) in characteristic path length (*p*_FWE_ = 0.0002, *k* = 23), clustering coefficient (*p*_FWE_ = 0.0001, *k* = 37) and modularity (*p*_FWE_ = 0.0001, *k* = 10). **D**+**E** Longitudinally, there was no evidence of group changes over time (TP3-TP1) in all three topological measures. *k* = empirical cluster size.

In the phase of severe underweight (TP1), patients with AN showed greater characteristic path length and modularity, and lower clustering coefficient compared with HC, indicating weaker network integration and segregation (Fig. 4A–C). Over time, there was no evidence of changes in characteristic path length, clustering coefficient, or modularity within either of the two groups (Fig. 4D+E). At the end of treatment (TP3), greater characteristic path length (AN: mean = 3.35 ± 0.48, HC: mean = 3.06 ± 0.57) and modularity (AN: mean = 0.39 ± 0.05, HC: mean = 0.37 ± 0.05) and lower clustering coefficient (AN: mean = 0.38 ± 0.06, HC: mean = 0.43 ± 0.09) persisted in patients with AN compared with HC (Fig. S1). Between TP2 and TP3, greater normalization of modularity correlated with earlier illness onset (*r* = 0.53, *p* = 0.0049, Holm-corrected *p* = 0.029). There was no evidence for associations of network topology with other clinical parameters or psychometric scores (see Supplemental Results).

## Discussion

We examined intrinsic network connectivity in women with severe AN over the course of weight-restoration treatment, compared with closely matched HC. Severely underweight patients with AN showed globally weaker and topologically altered intrinsic connectivity compared with HC, and network analysis revealed a widespread subnetwork of underconnectivity. Over the course of weight normalization, patients’ intrinsic connectivity within the subnetwork recovered only slightly, and network analysis at the end of treatment revealed a subnetwork of persistent underconnectivity in patients with AN. Consistent with these findings, significant differences in global strength and global network topology persisted after weight normalization at the end of treatment. Importantly, our results suggest that in adults with severe AN, short-term weight normalization may not be sufficient to fully restore intrinsic connectivity.

### Severe underweight

Results revealed globally weaker connectivity strength in women with severe AN, most strongly in a sizeable subnetwork of underconnectivity spanning large parts of the brain. Consistent with previous data-driven approaches [8, 10, 11, 20, 23, 24, 53, 54], we did not find evidence for overconnectivity in patients with severe AN. Results from graph metric analyses further clarified the network findings by showing that the global network topology of patients with severe AN is characterized by decreased network segregation (i.e., lower global clustering) and decreased network integration (i.e., greater characteristic path length and modularity). These findings substantiate previous reports of lower global clustering in adult patients [33] and greater characteristic path length in a younger sample of patients [9]. In line with previous reports [8, 11], there was no evidence that these differences were driven by alterations of regional homogeneity [55].

### Weight-dependent changes

This is the first longitudinal study to analyze intrinsic connectivity in severe AN with three measurement time points over the course of inpatient treatment. A major finding of the current study is that contrary to our hypothesis, there was no evidence for improvement of global connectivity strength and patients with AN continued to show a profile of underconnectivity despite weight normalization. Intrinsic connectivity within the weakened subnetwork of AN patients improved slightly during the first half of treatment (1–10 weeks after TP1), but weight normalization during the second half of treatment (11–20 weeks after TP1) did not further strengthen connectivity of the identified subnetwork. Additionally, there was no evidence for improvement of global network topology during weight normalization. Group comparisons at the end of treatment revealed strong differences compared with matched HC, indicating persistent underconnectivity and global topological alterations in patients with severe AN, independent of BMI.

Our results are consistent with reports of weaker inter‐network connectivity following weight normalization in adolescents and young adults [21] and support previous reports of persisting underconnectivity in long-term weight recovered patients with AN [22, 23, 56]. Divergent findings of rapid recovery of intrinsic connectivity after weight normalization [17, 20] could be due to differences in sample characteristics, namely a milder severity of illness at baseline [17] and a younger sample of patients with AN [20]. Another important factor could be differences in methodological approaches, with selective seed-to voxel approaches [17] possibly overlooking underconnectivity in other connections. The persisting topological alterations are in line with a first study reporting decreased network segregation in recovered patients [34], and further support the notion of altered intrinsic connectivity after weight-restoration. Importantly, these topological measures are thought to reflect key organizing principles of brain networks [57] and have been shown to predict cognitive performance in healthy individuals [58]. Taken together, our results provide new information by showing that in patients with severe AN, short-term weight normalization is not sufficient to restore the strength and topology of intrinsic connectivity.

### Clinical implications

In contrast to structural brain alterations, which have been show to quickly regenerate upon weight normalization in patients with AN [36, 59–61], intrinsic connectivity alterations in severe AN appear to be less influenced by weight normalization and thus do not solely represent a BMI-dependent state marker of AN. This is in line with findings from a large meta-analysis, reporting no correlation between resting-state brain activity and BMI [62]. Previous work has shown that weaker intrinsic connectivity is associated with more severe eating disorder symptoms, reflecting the altered cognitive patterns of patients with AN [63]. We hypothesize that the persisting brain alterations in patients with severe AN may be linked to the persisting cognitive symptoms of AN, as measured by the elevated EDE-Q scores at the end of treatment (TP3). Although no association between brain alterations and eating disorder symptoms was found in the present study, the patients’ persistent brain alterations and elevated eating disorder pathology scores after weight normalization emphasize that treatment success of severe AN should not be measured by BMI alone. Our findings underline the persistence of severe AN and point to the importance of continued psychotherapeutic support after inpatient treatment. Further investigation is needed to determine whether the persistent brain alterations are reversible with continued psychotherapeutic treatment and prolonged weight stabilization.

### Methodological considerations

Our findings have to be considered in the context of the following methodological considerations. First, preprocessing choices, such as brain parcellation [10, 64] and denoising strategies [65], can impact functional connectivity measures. Our findings are therefore likely contingent on the implemented parcellation scheme, which was chosen for its widespread usage in the field e.g., [60, 63, 64] to facilitate comparisons with other studies. To ensure the robustness of our findings with regard to motion correction, we repeated the preprocessing with a component based denoising method [66, 67]. The results indicated that the graph metrics of patients with AN were consistent with our initial findings (Supplemental Results), supporting their robustness.

Second, the question of how to handle negative connections (i.e., anticorrelations) is highly controversial and their meaning remains unclear, with some interpreting them as noise [68], while others see them as potentially meaningful connections [69]. For the present work, negative connections have been excluded since they have been shown to impair network reliability [70] and do not pertain to small-world properties [69]. Consequently, no conclusions regarding negative connections can be drawn from the present findings.

Third, while proportional thresholding is widely used and considered more reliable than absolute thresholding [71], differences in global strength may influence differences in graph metrics [72], since low-weight, possibly random, connections may be selected to meet the proportional density threshold [72, 73]. In weighted graphs, low-weight connections are presumed have a smaller effect on network topology [72], however, it should be noted that the investigated measures of global network topology inherently depend on connection strength and should be seen as complementing rather than independent findings.

Finally, although our sample size is well comparable to other brain imaging studies on AN it is nonetheless relatively moderate [74]. Evidently, larger cohorts are more sensitive to smaller effects and allow for a more precise estimation of larger effects. However, smaller studies can identify large clinically meaningful effects and, given the current lack of longitudinal resting-state data in AN, our study provides important insight to inform the hypotheses of future consortia studies. A strength of our study is the use of a reliable data-driven analysis method [47] that examines the whole brain without a priori restrictions to specific brain regions, while controlling the family-wise error rate. Furthermore, we accounted for the known sensitivity of intrinsic connectivity to natural fluctuations of anxiety during MRI scans [75] by collecting measurements of the HC group at two time points. Given the known substantial decrease of anxiety after first-time scans [76], this allowed for a more robust assessment of intrinsic connectivity in both groups.

## Conclusion

In conclusion, we have identified weakened intrinsic connectivity and disrupted network topology in patients with severe AN that were not improved by weight normalization over the course of specialized inpatient treatment. The persistent disruption of brain networks suggests sustained alterations of information processing in severe AN and underscores that body weight is only one part of the recovery.

## Supplementary information

Supplement
