# Supplementary material for: Disrupted longitudinal restoration of brain connectivity during weight normalization in severe anorexia nervosa
Source: Transl Psychiatry. 2023 Apr 28;13:136. doi: 10.1038/s41398-023-02428-z (PMC10147636; doi:10.1038/s41398-023-02428-z)
Supplement: Supplementary file 1 — Supplement [file 41398_2023_2428_MOESM1_ESM.docx]

**Disrupted longitudinal restoration of brain connectivity during weight normalization in severe anorexia nervosa**

***- Supplemental Information -***

**Contents**

[Supplemental Methods 2](#_Toc131425917)

[Participants 2](#_Toc131425918)

[Procedure 2](#_Toc131425919)

[MRI data acquisition 2](#_Toc131425920)

[MRI data preprocessing 3](#_Toc131425921)

[Correlation analyses 4](#_Toc131425922)

[Software 4](#_Toc131425923)

[Supplemental Results 8](#_Toc131425924)

[Subnetwork analyses 8](#_Toc131425925)

[Regional homogeneity 12](#_Toc131425926)

[Global network topology 14](#_Toc131425927)

[Alternative motion correction 14](#_Toc131425928)

[Correlation analyses 15](#_Toc131425929)

[Medication effects 20](#_Toc131425930)

[References 21](#_Toc131425931)

# Supplemental Methods

## Participants

Patients with anorexia nervosa (AN) receiving medication were instructed to continue them as prescribed to avoid treatment disturbances. Sixteen patients (59.26%) were taking psychotropic medication: antidepressants (8), atypical antipsychotics (1), antidepressants and atypical antipsychotics (3), antidepressants and anxiolytics (1), atypical antipsychotics and anxiolytics (1), antidepressants and atypical antipsychotics and anxiolytics (2). The Structured Clinical Interview for DSM-IV-TR [SCID-I, 1] was used to assess axis-I psychiatric disorders according to the DSM-IV-TR [2]. Comorbid diagnoses included major depressive disorder (11), major depressive disorder and social phobia (2), and obsessive-compulsive disorder (1). The majority of patients where amenorrhoeic (24, 85%), those with a cycle were part of the small subgroup taking hormonal contraceptives (8, 30%). All patients were examined at the Department of Internal Medicine and were hemodynamically stable.

## Procedure

Both groups underwent the same standardized intake procedure. Healthy controls were instructed to follow the same meal schedule as the patients on the day of scanning, i.e., have a regular lunch 2 hours before the appointment, no alcohol for 24 hours, and no caffeine prior to scanning.

## MRI data acquisition

A 3.0 Tesla whole-body magnetic resonance imaging (MRI) system (Ingenia, Philips Healthcare, Best, The Netherlands) equipped with a 32-channels receive phased array head coil was used for data acquisition. 3D T1-weighted structural images were acquired using a three-dimensional turbo field echo (TFE) sequence with echo time (TE)=3.8 ms, repetition time (TR)=8.3 ms, inversion time=1.0 s, field of view (FOV)=240×240 mm^2^, acquisition matrix=240×240, 160 sagittal slices, isotropic voxel size=1 mm^3^, flip angle=8°, and TFE factor=240 within 4.5 min. Resting-state functional images were acquired with eyes closed using a gradient-echo T2*-weighted echo planar imaging (EPI) sequence with TE=30 ms, TR=2.3 s, flip angle=78°, FOV=220×220×143 mm^3^, isotropic voxel size=3×3×3 mm^3^, 40 axial slices, EPI factor=41, and 210 volumes with a scan duration of ~8 min. For all subjects, T1-weighted and resting-state images were acquired and inspected by a trained neuroradiologist for any relevant pathology.

## MRI data preprocessing

Functional MRI data were processed with the DPARSFA toolbox (version 4.5, RRID:SCR_002372) as part of DPABI (version 4.2, RRID:SCR_010501) [3]. The first 5 volumes were discarded to allow for signal stabilization. The remaining 205 volumes were preprocessed using the following steps: 1) slice time correction, 2) realignment and extraction of mean frame-wise displacement values [4], 3) coregistration of the mean resting-state functional MRI with the T1-weighted image, 4) segmentation and normalization of the T1-weighted image to MNI152 structural space using linear and non-linear transformation, and storing the transformations, 5) application of the transformations from step 3 to the resting-state functional MRI data and voxel re-sampling to 2×2×2 mm^3^, 6) smoothing with an isotropic Gaussian kernel of 4 mm full width at half maximum (except for the regional homogeneity calculation), 7) detrending, 8) band-pass filtering between 0.01-0.08 Hz, and 9) regressing out the variance of head motion (24-parameter model) [5], the mean signals of cerebrospinal fluid and white matter, as well as framewise displacement (FD, defined as the root mean squared volume-to-volume displacement of all brain voxels measured from the six head motion parameters) [4] greater 0.5 mm, including the preceding and the two subsequent volumes, as recommended by Yan et al. [6]. This scrubbing approach within regression (i.e., spike regression [7]) was preferred to post-regression scrubbing (i.e., volume removal) since it has been shown to better account for motion-induced fluctuations in the BOLD signal [6].

### Head motion and quality control

There was no evidence for group differences in head motion (TP1: AN: mean FD = 0.12 mm ± 0.04 mm, HC: mean FD = 0.13 mm ± 0.07 mm, *p = .*59; TP3: AN: mean FD = 0.12 mm ± 0.04 mm, HC: mean FD = 0.13 mm ± 0.06 mm, *p = .*74) or number of suprathreshold frames considered for spike regression (TP1: AN: mean number of suprathreshold frames = 1.41 ± 2.89, HC: mean number of suprathreshold frames = 3.93 ± 9.24, *p = .*11; TP3: AN: mean number of suprathreshold frames = 2.04 ± 3.01, HC: mean number of suprathreshold frames = 2.75 ± 8.86, *p = .*64). Additionally, there was no evidence of a relationship between the connectivity measures (mean global strength, mean subnetwork connectivity, mean characteristic path length, mean clustering coefficient, and mean modularity) and measures of motion (mean FD, number of suprathreshold frames) (all *p* > 0.05).

### Global signal regression

Global signal was not regressed out during preprocessing, as this step has been shown to lower test-retest reliability [6] and is known to increase anti-correlations and distance-dependent artefacts [8], which are problematic for network-based analyses.

### Parcellation scheme

Ninety regions of the automated anatomical labelling (AAL) atlas were used to delineate the network nodes [9]. The insular cortex of both hemispheres was divided into its anterior and posterior parts, as it has been shown that the two regions are associated with distinct brain functions and are involved in dissociable resting-state networks. The functional segmentation of the insula in its anterior and posterior parts based on resting-state fMRI data of an independent sample was performed in a previous study of our group and is described in detail elsewhere [10]. Additionally, the nucleus accumbens (derived from the Harvard-Oxford subcortical atlas, [11]) was added for both hemispheres, as this structure is of interest in AN research due to its involvement in reward processes [12], adding up to a total number of 94 regions used for the brain parcellation during preprocessing (see **Table S1**).

## Correlation analyses

Pearson correlations were calculated to test for associations between measures of functional connectivity and clinical (BMI, age, age of onset, illness duration) or psychometric parameters (EDE-Q, BDI), using R (version 4.2.2, RRID:SCR_001905) [13]. Reported *p-*values are adjusted for multiple comparisons using the Holm−Bonferroni procedure [14].

## Software

The following R-packages were used for data wrangling, analyses, and visualizations within R (version 4.2.2, RRID:SCR_001905) [13]: *broom* [15], *corrplot* [16], *cowplot* [17], *dplyr* [18], *forcats* [19], *GGally* [20], *ggplot2* [21], *ggraph* [22], *gridExtra* [23], *gtsummary* [24], *igraph* [25], *lubridate* [26], *NetworkToolbox* [27], *patchwork* [28], *psych* [29], *purrr* [30], *RColorBrewer* [31], *readr* [32], *readxl* [33], *rlang* [34], *rstatix* [35], *stringr* [36], *tibble* [37], *tidyr* [38], *tidyverse* [39].

**Table S1**

*Parcellation scheme with 94 regions*

| Number | Anatomical description | Number | Anatomical description |
| --- | --- | --- | --- |
| 1 | Precentral gyrus (L) | 48 | Superior occipital gyrus (L) |
| 2 | Precentral gyrus (R) | 49 | Superior occipital gyrus (R) |
| 3 | Superior frontal gyrus, dorsolateral (L) | 50 | Middle occipital gyrus (L) |
| 4 | Superior frontal gyrus, dorsolateral (R) | 51 | Middle occipital gyrus (R) |
| 5 | Superior frontal gyrus, orbital part (L) | 52 | Inferior occipital gyrus (L) |
| 6 | Superior frontal gyrus, orbital part (R) | 53 | Inferior occipital gyrus (R) |
| 7 | Middle frontal gyrus (L) | 54 | Fusiform gyrus (L) |
| 8 | Middle frontal gyrus (R) | 55 | Fusiform gyrus (R) |
| 9 | Middle frontal gyrus, orbital part (L) | 56 | Postcentral gyrus (L) |
| 10 | Middle frontal gyrus, orbital part (R) | 57 | Postcentral gyrus (R) |
| 11 | Inferior frontal gyrus, opercular part (L) | 58 | Superior parietal gyrus (L) |
| 12 | Inferior frontal gyrus, opercular part (R) | 59 | Superior parietal gyrus (R) |
| 13 | Inferior frontal gyrus, triangular part (L) | 60 | Inferior parietal gyrus (L) |
| 14 | Inferior frontal gyrus, triangular part (R) | 61 | Inferior parietal gyrus (R) |
| 15 | Inferior frontal gyrus, orbital part (L) | 62 | Posterior cingulate gyrus (L) |
| 16 | Inferior frontal gyrus, orbital part (R) | 63 | Posterior cingulate gyrus (R) |
| 17 | Rolandic operculum (L) | 64 | Hippocampus (L) |
| 18 | Rolandic operculum (R) | 65 | Hippocampus (R) |
| 19 | Supplementary motor area (L) | 66 | Parahippocampus (L) |
| 20 | Supplementary motor area (R) | 67 | Paracentral lobule (L) |
| 21 | Olfactory cortex (L) | 68 | Paracentral lobule (R) |
| 22 | Olfactory cortex (R) | 69 | Caudate nucleus (L) |
| 23 | Superior frontal gyrus, medial (L) | 70 | Caudate nucleus (R) |
| 24 | Superior frontal gyrus, medial (R) | 71 | Putamen (L) |
| 25 | Superior frontal gyrus, medial orbital (L) | 72 | Putamen (R) |
| 26 | Superior frontal gyrus, medial orbital (R) | 73 | Pallidum (L) |
| 27 | Gyrus rectus (L) | 74 | Pallidum (R) |
| 28 | Gyrus rectus (R) | 75 | Thalamus (L) |
| 29 | Anterior cingulate & paracingulate gyri (L) | 76 | Thalamus (R) |
| 30 | Anterior cingulate & paracingulate gyri (R) | 77 | Heschl's gyrus (L) |
| 31 | Middle cingulate & paracingulate gyri (L) | 78 | Heschl's gyrus (R) |
| 32 | Middle cingulate & paracingulate gyri (R) | 79 | Superior temporal gyrus (L) |
| 33 | Precentral gyrus (L) | 80 | Superior temporal gyrus (R) |
| 34 | Posterior cingulate gyrus (L) | 81 | Temporal pole: superior temporal gyrus (L) |
| 35 | Posterior cingulate gyrus (R) | 82 | Temporal pole: superior temporal gyrus (R) |
| 36 | Hippocampus (L) | 83 | Middle temporal gyrus (L) |
| 37 | Hippocampus (R) | 84 | Middle temporal gyrus (R) |
| 38 | Parahippocampus (L) | 85 | Temporal pole: middle temporal gyrus (L) |
| 39 | Parahippocampus (R) | 86 | Temporal pole: middle temporal gyrus (R) |
| 40 | Amygdala (L) | 87 | Inferior temporal gyrus (L) |
| 41 | Amygdala (R) | 88 | Inferior temporal gyrus (R) |
| 42 | Calcarine ﬁssure and surrounding cortex (L) | 89 | Nucleus accumbens (L) |
| 43 | Calcarine ﬁssure and surrounding cortex (R) | 90 | Nucleus accumbens (R) |
| 44 | Cuneus (L) | 91 | Insula anterior (L) |
| 45 | Cuneus (R) | 92 | Insula anterior (R) |
| 46 | Lingual gyrus (L) | 93 | Insula posterior (L) |
| 47 | Lingual gyrus (R) | 94 | Insula posterior (R) |
| *Note.* Regions 89 to 94 were added to the automated anatomical labelling atlas. L = left hemisphere, R = right hemisphere. | | | |

# Supplemental Results

## Subnetwork analyses

### Subnetworks

At TP1, NBS analysis yielded a subnetwork of underconnectivity in patients with AN compared with HC (*p* < 0.004, FWE-corrected). The subnetwork comprised 61 nodes and 125 connections, listed in **Table S2**. At TP3, NBS analysis yielded a slightly smaller subnetwork of underconnectivity in patients with AN compared with HC (*p* < 0.004, FWE-corrected). The subnetwork comprised 33 nodes and 48 connections, listed in **Table S3**. Regarding longitudinal changes, NBS analyses within the AN group between TP1 and TP2, and between TP2 and TP3, yielded no evidence of subnetworks changing over the course of weight normalization. Similarly, NBS analysis of both groups (group × time) yielded no evidence of network changes over the course of treatment.

### Alternative primary thresholds

To assess the robustness of results with alternative primary thresholds, the group comparisons at TP1 and TP3 were repeated with primary thresholds of *t* = 2.4 and *t* = 3.2 (corresponding to *p* = 0.01 and *p* = 0.001). Our findings of underconnectivity in patients with AN were replicated, with the size of the identified subnetworks varying as expected, with larger subnetworks of underconnectivity at *t* = 2.4 (TP1: 83 nodes and 364 connections, *p* < 0.007, FWE-corrected; TP3: 72 nodes and 229 connections, *p* < 0.025, FWE-corrected) and smaller subnetworks of underconnectivity at *t* = 3.2 (TP1: 54 nodes and 96 connections, *p* < 0.004, FWE-corrected; TP3: 19 nodes and 20 connections, *p* < 0.05, FWE-corrected). Within the AN group, the absence of subnetworks changing over the course of weight normalization was replicated with more lenient primary thresholds of *t* = 0.6 and *t* = 1.7 (corresponding to *p* < 0.30 and *p* < 0.05; no components detected). Further, our findings of no evidence of change between groups over time (group × time) were replicated with very lenient primary thresholds of *F* = 0.5 and *F* = 1.0 (corresponding to *p* < 0.50 and *p* = 0.32).

**Table S2**

*Connections of the subnetwork of underconnectivity in patients with AN at TP1*

| Node A | Node B | Node A | Node B |
| --- | --- | --- | --- |
| Nucleus accumbens (R) | Insula anterior (L) | Fusiform gyrus (L) | Superior temporal gyrus (L) |
| Amygdala (R) | Putamen (L) | Fusiform gyrus (L) | Middle temporal gyrus (L) |
| Calcarine (L) | Putamen (L) | Fusiform gyrus (L) | Insula posterior (R) |
| Calcarine (L) | Pallidum (L) | Fusiform gyrus (R) | Postcentral (L) |
| Calcarine (R) | Putamen (L) | Fusiform gyrus (R) | Putamen (L) |
| Calcarine (R) | Pallidum (L) | Fusiform gyrus (R) | Putamen (R) |
| Anterior cingulate & paracingulate gyri (L) | Putamen (L) | Fusiform gyrus (R) | Heschl’s gyrus (L) |
| Anterior cingulate & paracingulate gyri (L) | Pallidum (L) | Fusiform gyrus (R) | Insula posterior (L) |
| Anterior cingulate & paracingulate gyri (R) | Pallidum (L) | Fusiform gyrus (R) | Insula posterior (R) |
| Cuneus (R) | Putamen (L) | Hippocampus (L) | Caudate nucleus (L) |
| Inferior frontal gyrus, opercular (L) | Pallidum (L) | Hippocampus (L) | Nucleus accumbens (R) |
| Inferior frontal gyrus, opercular (L) | Pallidum (R) | Hippocampus (R) | Putamen (L) |
| Inferior frontal gyrus, opercular (R) | Putamen (R) | Hippocampus (R) | Nucleus accumbens (L) |
| Inferior frontal gyrus, opercular (R) | Pallidum (L) | Hippocampus (R) | Nucleus accumbens (R) |
| Inferior frontal gyrus, opercular (R) | Pallidum (R) | Lingual gyrus (L) | Putamen (L) |
| Inferior frontal gyrus, orbital (L) | Middle occipital lobe (R) | Lingual gyrus (L) | Putamen (R) |
| Inferior frontal gyrus, triangular (L) | Pallidum (R) | Lingual gyrus (L) | Pallidum (L) |
| Inferior frontal gyrus, triangular (R) | Pallidum (R) | Lingual gyrus (R) | Putamen (L) |
| Middle frontal gyrus (L) | Pallidum (R) | Lingual gyrus (R) | Pallidum (L) |
| Middle frontal gyrus (R) | Fusiform gyrus (L) | Inferior occipital gyrus (L) | Postcentral (R) |
| Middle frontal gyrus (R) | Middle temporal gyrus (L) | Inferior occipital gyrus (L) | Putamen (R) |
| Middle frontal gyrus (R) | Inferior temporal gyrus (L) | Inferior occipital gyrus (L) | Pallidum (L) |
| Superior frontal gyrus, dorsolateral (L) | Inferior occipital gyrus (R) | Inferior occipital gyrus (L) | Pallidum (R) |
| Superior frontal gyrus, dorsolateral (L) | Pallidum (R) | Inferior occipital gyrus (R) | Postcentral (L) |
| Superior frontal gyrus, medial (L) | Middle temporal gyrus (R) | Inferior occipital gyrus (R) | Supra Marginal (L) |
| Superior frontal gyrus, medial orbital (L) | Olfactory (L) | Inferior occipital gyrus (R) | Paracentral lobule (L) |
| Superior frontal gyrus, medial orbital (L) | Middle occipital lobe (R) | Inferior occipital gyrus (R) | Putamen (L) |
| Superior frontal gyrus, dorsolateral (R) | Putamen (L) | Inferior occipital gyrus (R) | Pallidum (L) |
| Superior frontal gyrus, dorsolateral (R) | Middle temporal gyrus (L) | Inferior occipital gyrus (R) | Pallidum (R) |
| Fusiform gyrus (L) | Postcentral (R) | Inferior occipital gyrus (R) | Heschl’s gyrus (L) |
| Inferior occipital gyrus (R) | Superior temporal gyrus (L) | Parahippocampus (R) | Putamen (L) |
| Inferior occipital gyrus (R) | Superior temporal gyrus (R) | Postcentral (R) | Inferior temporal gyrus (L) |
| Inferior occipital gyrus (R) | Middle temporal gyrus (R) | Precentral (L) | Inferior occipital gyrus (R) |
| Inferior occipital gyrus (R) | Insula posterior (L) | Precentral (L) | Fusiform gyrus (R) |
| Middle occipital lobe (L) | Caudate nucleus (L) | Precentral (L) | Superior temporal gyrus (R) |
| Middle occipital lobe (L) | Caudate nucleus (R) | Precentral (R) | Inferior occipital gyrus (L) |
| Middle occipital lobe (L) | Putamen (L) | Precentral (R) | Inferior occipital gyrus (R) |
| Middle occipital lobe (L) | Pallidum (L) | Precentral (R) | Fusiform gyrus (L) |
| Middle occipital lobe (L) | Pallidum (R) | Precentral (R) | Inferior temporal gyrus (L) |
| Middle occipital lobe (L) | Nucleus accumbens (L) | Precuneus (R) | Caudate nucleus (L) |
| Middle occipital lobe (L) | Nucleus accumbens (R) | Putamen (L) | Pallidum (R) |
| Middle occipital lobe (R) | Caudate nucleus (L) | Putamen (L) | Heschl’s gyrus (R) |
| Middle occipital lobe (R) | Putamen (L) | Putamen (L) | Superior temporal gyrus (R) |
| Middle occipital lobe (R) | Pallidum (L) | Putamen (L) | Middle temporal gyrus (R) |
| Middle occipital lobe (R) | Pallidum (R) | Putamen (L) | Nucleus accumbens (L) |
| Middle occipital lobe (R) | Middle temporal gyrus (L) | Putamen (L) | Nucleus accumbens (R) |
| Middle occipital lobe (R) | Nucleus accumbens (L) | Putamen (R) | Heschl’s gyrus (L) |
| Middle occipital lobe (R) | Insula anterior (L) | Putamen (R) | Heschl’s gyrus (R) |
| Superior occipital lobe (L) | Nucleus accumbens (L) | Putamen (R) | Superior temporal gyrus (L) |
| Superior occipital lobe (R) | Caudate nucleus (L) | Putamen (R) | Superior temporal gyrus (R) |
| Superior occipital lobe (R) | Putamen (L) | Putamen (R) | Insula posterior (R) |
| Superior occipital lobe (R) | Pallidum (L) | Rolandic operculum (L) | Pallidum (R) |
| Superior occipital lobe (R) | Nucleus accumbens (L) | Rolandic operculum (R) | Inferior occipital gyrus (L) |
| Superior occipital lobe (R) | Insula anterior (L) | Rolandic operculum (R) | Inferior occipital gyrus (R) |
| Olfactory (L) | Superior occipital lobe (R) | Rolandic operculum (R) | Fusiform gyrus (L) |
| Olfactory (L) | Middle occipital lobe (R) | Rolandic operculum (R) | Fusiform gyrus (R) |
| Olfactory (R) | Middle occipital lobe (L) | Rolandic operculum (R) | Pallidum (L) |
| Pallidum (R) | Heschl’s gyrus (L) | Rolandic operculum (R) | Pallidum (R) |
| Pallidum (R) | Heschl’s gyrus (R) | Supplementary motor area (L) | Inferior occipital gyrus (R) |
| Pallidum (R) | Superior temporal gyrus (L) | Supplementary motor area (R) | Inferior occipital gyrus (L) |
| Pallidum (R) | Superior temporal gyrus (R) | Supplementary motor area (R) | Inferior occipital gyrus (R) |
| Pallidum (R) | Insula posterior (L) | Middle temporal gyrus (L) | Inferior temporal gyrus (L) |
| Pallidum (R) | Insula posterior (R) |  |  |
| *Note.* Connections represent reduced functional connectivity between regions (node A and node B) in patients with anorexia nervosa, compared to healthy controls at time point 1 (TP1, *t*=3.0, p < 0.004, FWE-corrected). L = left hemisphere, R = right hemisphere. | | | |

**Table S3**

*Connections of the subnetwork of underconnectivity in patients with AN at TP3*

| Node A | Node B | Node A | Node B |
| --- | --- | --- | --- |
| Heschl’s gyrus (R) | Superior temporal gyrus (L) | Rolandic operculum (R) | Supplementary motor area (L) |
| Heschl’s gyrus (R) | Middle temporal gyrus (L) | Rolandic operculum (R) | Supplementary motor area (R) |
| Heschl’s gyrus (R) | Middle temporal gyrus (R) | Rolandic operculum (R) | Superior occipital lobe (L) |
| Inferior occipital lobe (L) | Postcentral gyrus (R) | Rolandic operculum (R) | Middle occipital lobe (L) |
| Inferior occipital lobe (L) | Supramarginal gyrus (R) | Rolandic operculum (R) | Middle occipital lobe (R) |
| Inferior occipital lobe (L) | Paracentral lobule (L) | Rolandic operculum (R) | Inferior parietal gyrus (L) |
| Inferior occipital lobe (L) | Paracentral lobule (R) | Rolandic operculum (R) | Precuneus (L) |
| Inferior occipital lobe (R) | Supramarginal gyrus (L) | Rolandic operculum (R) | Superior temporal gyrus (R) |
| Inferior occipital lobe (R) | Paracentral lobule (L) | Rolandic operculum (R) | Middle temporal gyrus (L) |
| Inferior occipital lobe (R) | Temporal pole: middle temporal gyrus (R) | Superior frontal gyrus, orbital (L) | Superior occipital lobe (R) |
| Inferior parietal gyrus (L) | Superior temporal gyrus (R) | Superior frontal gyrus, orbital (L) | Middle occipital lobe (L) |
| Middle frontal gyrus, orbital (L) | Middle occipital lobe (L) | Superior frontal gyrus, orbital (R) | Superior temporal gyrus (R) |
| Middle frontal gyrus, orbital (L) | Inferior occipital lobe (R) | Superior frontal gyrus, orbital (R) | Cuneus (R) |
| Middle occipital lobe (L) | Superior temporal gyrus (R) | Superior frontal gyrus, orbital (R) | Superior occipital lobe (L) |
| Middle occipital lobe (L) | Temporal pole: middle temporal gyrus (R) | Superior frontal gyrus, orbital (R) | Middle occipital lobe (L) |
| Middle occipital lobe (R) | Temporal pole: middle temporal gyrus (L) | Superior frontal gyrus, orbital (R) | Inferior occipital lobe (R) |
| Middle temporal gyrus (L) | Inferior temporal gyrus (L) | Superior occipital lobe (L) | Superior temporal gyrus (R) |
| Postcentral (L) | Superior temporal gyrus (R) | Superior temporal gyrus (R) | Temporal pole: middle temporal gyrus (L) |
| Postcentral gyrus (R) | Precuneus (R) | Supplementary motor area (L) | Heschl’s gyrus (R) |
| Postcentral gyrus (R) | Middle temporal gyrus (L) | Supplementary motor area (L) | Superior temporal gyrus (R) |
| Postcentral gyrus (R) | Inferior temporal gyrus (L) | Supplementary motor area (R) | Inferior occipital lobe (L) |
| Precentral (R) | Middle temporal gyrus (L) | Supplementary motor area (R) | Superior temporal gyrus (R) |
| Precentral gyrus (L) | Superior temporal gyrus (R) | Supramarginal gyrus (R) | Inferior temporal gyrus (L) |
| Precuneus (R) | Heschl’s gyrus (R) | Temporal pole: middle temporal gyrus (L) | Insula posterior (R) |
| *Note.* Connections represent reduced functional connectivity between regions in patients with anorexia nervosa, compared to healthy controls at time point 3 (TP3, *t*=3.0, p < 0.004, FWE-corrected). L = left hemisphere, R = right hemisphere. | | | |

## Regional homogeneity

There was no evidence for group differences of regional homogeneity at TP1 (**Table S4**). Furthermore, exploratory group comparisons of correlations between regional homogeneity and inter-regional connectivity yielded no significant differences in correlations (all *p* > 0.05). This result is in line with a previous study in which alterations in functional connectivity were unrelated to local neuronal synchrony of patients with AN [40].

**Table S4**

*Group comparisons of regional homogeneity (TP1)*

|  | AN  *n* = 27 | | HC  *n* = 40 | |
| --- | --- | --- | --- | --- |
| Node | Mean | *SD* | Mean | *SD* |
| Nucleus accumbens (L) | -0.30 | 0.44 | -0.24 | 0.48 |
| Nucleus accumbens (R) | -0.31 | 0.57 | -0.26 | 0.56 |
| Amygdala (R) | -0.44 | 0.40 | -0.40 | 0.31 |
| Calcarine (L) | 0.69 | 0.37 | 0.72 | 0.27 |
| Calcarine (R) | 0.71 | 0.32 | 0.69 | 0.26 |
| Caudate nucleus (L) | -0.60 | 0.22 | -0.57 | 0.29 |
| Caudate nucleus (R) | -0.55 | 0.22 | -0.51 | 0.20 |
| Anterior cingulate & paracingulate gyri (L) | 0.01 | 0.26 | 0.11 | 0.24 |
| Anterior cingulate & paracingulate gyri (R) | -0.09 | 0.19 | -0.18 | 0.23 |
| Cuneus (R) | 0.54 | 0.39 | 0.61 | 0.31 |
| Inferior frontal gyrus, opercular (L) | 0.19 | 0.21 | 0.14 | 0.24 |
| Inferior frontal gyrus, opercular (R) | 0.10 | 0.23 | 0.03 | 0.22 |
| Inferior frontal gyrus, orbital (L) | -0.09 | 0.22 | -0.12 | 0.20 |
| Inferior frontal gyrus, triangular (L) | 0.22 | 0.27 | 0.12 | 0.26 |
| Inferior frontal gyrus, triangular (R) | 0.14 | 0.26 | 0.07 | 0.28 |
| Middle frontal gyrus (L) | 0.20 | 0.26 | 0.27 | 0.19 |
| Middle frontal gyrus (R) | 0.23 | 0.22 | 0.21 | 0.24 |
| Superior frontal gyrus, dorsolateral (L) | -0.01 | 0.24 | 0.03 | 0.19 |
| Superior frontal gyrus, medial (L) | -0.01 | 0.24 | 0.03 | 0.18 |
| Superior frontal gyrus, medial orbital (L) | -0.16 | 0.19 | -0.31 | 0.28 |
| Superior frontal gyrus, dorsolateral (R) | 0.03 | 0.21 | 0.02 | 0.19 |
| Fusiform gyrus (L) | -0.26 | 0.19 | -0.22 | 0.18 |
| Fusiform gyrus (R) | -0.22 | 0.21 | -0.18 | 0.23 |
| Heschl’s gyrus (L) | 0.14 | 0.48 | 0.14 | 0.46 |
| Heschl’s gyrus (R) | -0.03 | 0.42 | 0.16 | 0.35 |
| Hippocampus (L) | -0.25 | 0.34 | -0.35 | 0.32 |
| Hippocampus (R) | -0.52 | 0.26 | -0.46 | 0.27 |
| Insula anterior (L) | 0.08 | 0.32 | -0.02 | 0.24 |
| Insula posterior (L) | 0.18 | 0.40 | 0.13 | 0.34 |
| Insula posterior (R) | 0.02 | 0.28 | 0.07 | 0.30 |
| Lingual gyrus (L) | 0.56 | 0.37 | 0.53 | 0.30 |
| Lingual gyrus (R) | 0.55 | 0.36 | 0.55 | 0.32 |
| Inferior occipital gyrus (L) | 0.26 | 0.38 | 0.25 | 0.36 |
| Inferior occipital gyrus (R) | 0.36 | 0.54 | 0.38 | 0.42 |
| Middle occipital lobe (L) | 0.33 | 0.26 | 0.34 | 0.24 |
| Middle occipital lobe (R) | 0.45 | 0.27 | 0.43 | 0.20 |
| Superior occipital lobe (L) | 0.33 | 0.32 | 0.48 | 0.25 |
| Superior occipital lobe (R) | 0.47 | 0.26 | 0.47 | 0.20 |
| Olfactory (L) | -0.64 | 0.31 | -0.67 | 0.36 |
| Olfactory (R) | -0.69 | 0.32 | -0.67 | 0.35 |
| Pallidum (L) | -0.22 | 0.39 | -0.21 | 0.29 |
| Pallidum (R) | -0.30 | 0.48 | -0.18 | 0.31 |
| Paracentral lobule (L) | 0.11 | 0.34 | 0.16 | 0.34 |
| Parahippocampus (R) | -0.69 | 0.25 | -0.58 | 0.24 |
| Postcentral gyrus (L) | 0.17 | 0.21 | 0.25 | 0.26 |
| Postcentral gyrus (R) | 0.08 | 0.25 | 0.22 | 0.29 |
| Precentral gyrus (L) | 0.01 | 0.22 | 0.05 | 0.18 |
| Precentral gyrus (R) | -0.08 | 0.24 | 0.08 | 0.26 |
| Precuneus (R) | 0.62 | 0.18 | 0.55 | 0.20 |
| Putamen (L) | -0.22 | 0.20 | -0.14 | 0.26 |
| Putamen (R) | -0.24 | 0.22 | -0.16 | 0.26 |
| Rolandic operculum (L) | 0.09 | 0.35 | 0.04 | 0.39 |
| Rolandic operculum (R) | 0.01 | 0.26 | 0.01 | 0.23 |
| Supplementary motor area (L) | 0.10 | 0.23 | 0.20 | 0.21 |
| Supplementary motor area (R) | 0.04 | 0.28 | 0.03 | 0.22 |
| Supramarginal gyrus (L) | 0.33 | 0.29 | 0.26 | 0.27 |
| Inferior temporal gyrus (L) | -0.19 | 0.24 | -0.24 | 0.25 |
| Middle temporal gyrus (L) | 0.12 | 0.20 | 0.11 | 0.22 |
| Middle temporal gyrus (R) | 0.14 | 0.23 | 0.10 | 0.27 |
| Superior temporal gyrus (L) | 0.16 | 0.24 | 0.17 | 0.28 |
| Superior temporal gyrus (R) | 0.04 | 0.23 | -0.24 | 0.48 |
| *Notes.* Regional homogeneity: no evidence for group differences (all *p* > 0.05, two-tailed, Holm-corrected). *SD* = standard deviation. | | | | |

## Global network topology

### Residual alterations

After weight normalization (TP3), greater characteristic path length (AN: mean = 3.35 ± 0.48, HC: mean = 3.06 ± 0.57) and modularity (AN: mean = 0.39 ± 0.05, HC: mean = 0.37 ± 0.05) and lower clustering coefficient (AN: mean = 0.38 ± 0.06, HC: mean = 0.43 ± 0.09) persisted in patients with AN compared with HC (Fig. S1).

| 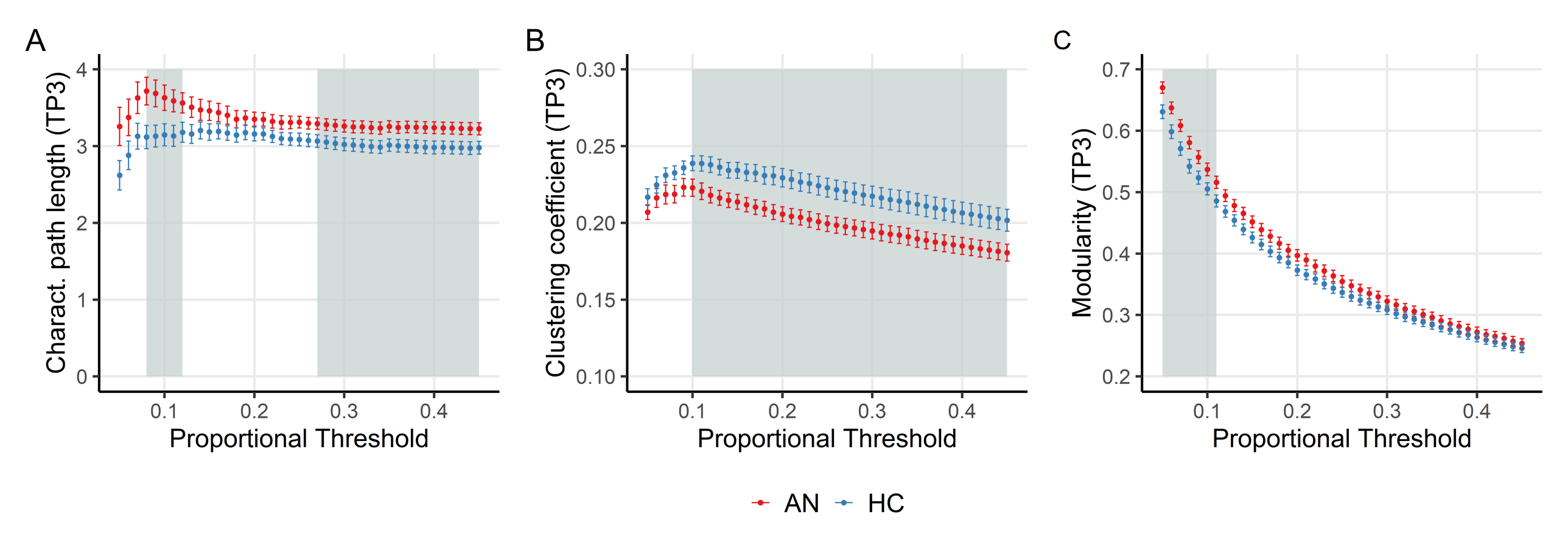 |
| --- |
| **Figure S1.** Global network topology compared between patients with AN (red) and healthy controls (blue) at TP3. Cluster-based permutation testing revealed persisting group differences (gray-shaded areas) in characteristic path length (*p*_FWE_ = 0.0001, *k_1_* = 5, *k_2_* = 19), clustering coefficient (*p*_FWE_ = 0.0001, *k* = 36) and modularity (*p*_FWE_ = 0.0001, *k* = 7). *k* = empirical cluster size. |

## Alternative motion correction

To ensure the robustness of our findings, we reran the MRI data preprocessing using aCompCor [41, 42] for motion correction. This approach included the use of the first five principal components for cerebrospinal fluid and white matter as motion regressors to minimize any potential impact of motion on the results. The comparison of the two preprocessing approaches (spike regression, aCompCor) showed a substantial degree of overlap between the distributions for all measures for patients with AN at TP1 (Fig. S2). Additionally, paired two-tailed Welch’s *t*-tests showed no evidence for statistically significant differences in any of the measures (mean global strength: *t*(26) = 0.227, *p* = .822, *g* = -0.042; mean characteristic path length: *t*(26) = 0.547, *p* = .589, *g* = -0.102; mean clustering coefficient: *t*(26) = 0.051, *p* = .960, *g* = -0.010; mean modularity: *t*(26) = 0.594, *p* = .558, *g* = -0.111). This further supports the robustness of our findings and shows that the conclusions of our study are not likely dependent on the specific denoising method used.

| 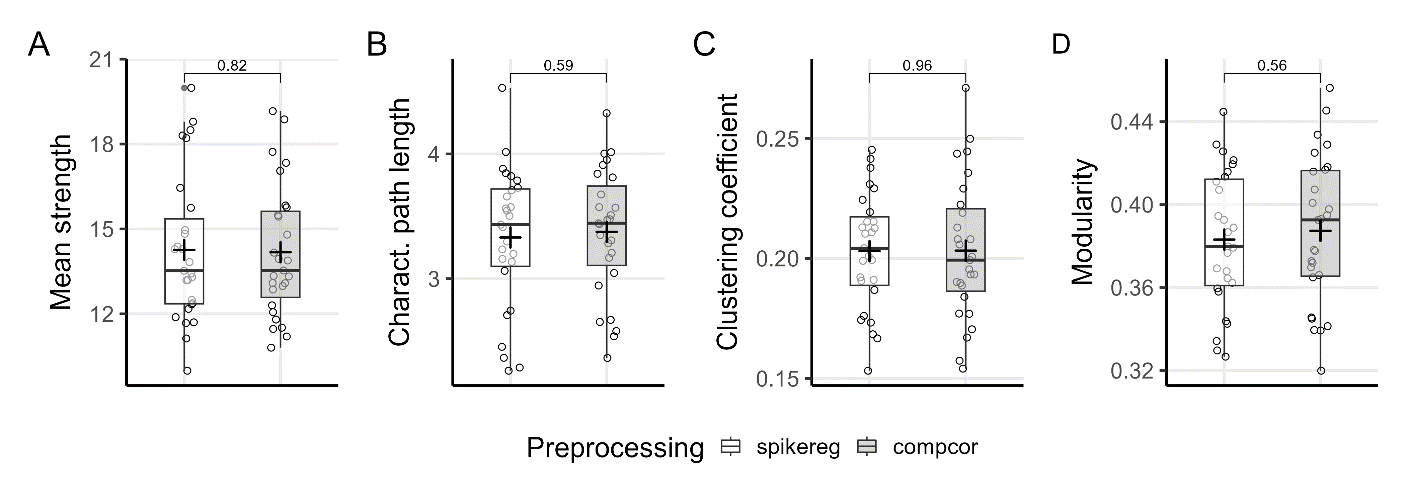 |
| --- |
| **Figure S2.** Boxplots comparing graph metrics between two different motion correction approaches during preprocessing for patients with AN at TP1. The cross signifies the mean, the horizontal mark signifies the median, edges of the box represent 25th and 75th percentiles, and the whiskers extend to 1.5 interquartile ranges. Values above the boxplots show the p-values per comparison, resulting from paired Welch’s *t*-tests between the two motion correction procedures: compcor = aCompCor, spikereg = spike regression. |

## Correlation analyses

To explore whether individual differences in clinical (BMI, age, age at illness onset, illness duration) or psychometric measures (EDE-Q, BDI) would explain changes in connectivity, correlations within the patient group were calculated for measures at TP1 and for change scores in the early (between TP1 and TP2) and the later treatment phase (between TP2 and TP3). Reduced global strength, underconnectivity of the subnetwork at TP1, and altered graph metrics were not explained by clinical or psychometric measures (**Table S5**).

**Table S5**

*Correlations between connectivity measures* *and clinical or psychometric measures within the group of patients with anorexia nervosa*

|  |  | AN  *n* = 27 | |  |
| --- | --- | --- | --- | --- |
| Connectivity measures | Clinical / psychometric measures | *r* | *p* |  |
| Global strength (TP1) | BMI (TP1) | -0.39 | 0.05 |  |
|  | Age | 0.13 | 0.51 |  |
|  | Age at onset | 0.33 | 0.09 |  |
|  | Illness duration | -0.12 | 0.55 |  |
|  | EDE-Q total (TP1) | 0.07 | 0.75 |  |
|  | BDI (TP1) | 0.04 | 0.87 |  |
| Subnetwork connectivity (TP1) | BMI (TP1) | -0.14 | 0.49 |  |
|  | Age | 0.08 | 0.68 |  |
|  | Age at onset | 0.25 | 0.21 |  |
|  | Illness duration | -0.10 | 0.64 |  |
|  | EDE-Q total (TP1) | -0.13 | 0.52 |  |
|  | BDI (TP1) | -0.13 | 0.51 |  |
| Characteristic path length (TP1) | BMI (TP1) | 0.34 | 0.09 |  |
|  | Age | -0.16 | 0.41 |  |
|  | Age at onset | -0.24 | 0.22 |  |
|  | Illness duration | 0.04 | 0.86 |  |
|  | EDE-Q total (TP1) | -0.13 | 0.54 |  |
|  | BDI (TP1) | -0.08 | 0.71 |  |
| Clustering coefficient (TP1) | BMI (TP1) | -0.10 | 0.61 |  |
|  | Age | 0.25 | 0.20 |  |
|  | Age at onset | 0.30 | 0.14 |  |
|  | Illness duration | 0.02 | 0.92 |  |
|  | EDE-Q total (TP1) | 0.03 | 0.88 |  |
|  | BDI (TP1) | -0.06 | 0.78 |  |
| Modularity (TP1) | BMI (TP1) | 0.23 | 0.24 |  |
|  | Age | -0.20 | 0.33 |  |
|  | Age at onset | -0.15 | 0.45 |  |
|  | Illness duration | -0.08 | 0.71 |  |
|  | EDE-Q total (TP1) | -0.46 | 0.02 |  |
|  | BDI (TP1) | -0.16 | 0.42 |  |
| *Notes.* Subnetwork connectivity of the subnetwork identified at TP1. BDI = Beck Depression Inventory; EDE-Q = Eating disorder examination questionnaire; TP = time point. No statistically significant correlations after Holm correction. | | | | |

In terms of longitudinal changes, there was no evidence of a relationship between intrinsic connectivity and clinical measures in the early treatment phase (**Table S6**). In the later treatment phase, greater normalization of modularity correlated with earlier illness onset (modularity: *r* = 0.53, *p* = 0.0049, Holm-corrected *p* = 0.029), possibly suggesting an interaction of illness onset and developmental processes of network maturation [43, 44]. Similarly, normalization of global strength and characteristic path length during the later treatment phase correlated with older age at illness onset (global strength: *r* = -0.46, *p* = 0.016, Holm-corrected *p* = 0.096; characteristic path length: *r* = 0.42 *p* = 0.029, Holm-corrected *p* = 0.172), but these associations did not remain statistically significant after correction for multiple comparisons. There was no evidence for associations of connectivity with psychometric scores in any of the treatment phases (**Table S6**).

**Table S6**

*Correlations between change scores of connectivity measures and clinical or psychometric measures within the group of patients with anorexia nervosa*

|  |  | AN  *n* = 27 | |  |
| --- | --- | --- | --- | --- |
| Connectivity measures | Clinical / psychometric measures | *r* | *p* |  |
| Global strength (TP2-1) | BMI (TP2-1) | 0.03 | 0.86 |  |
|  | Age | 0.19 | 0.35 |  |
|  | Age at onset | 0.10 | 0.63 |  |
|  | Illness duration | 0.13 | 0.52 |  |
|  | EDE-Q total (TP2-1) | -0.04 | 0.83 |  |
|  | BDI (TP2-1) | -0.31 | 0.14 |  |
| Global strength (TP3-2) | BMI (TP3-2) | -0.38 | 0.05 |  |
|  | Age | -0.21 | 0.29 |  |
|  | Age at onset | -0.46 | 0.02 |  |
|  | Illness duration | 0.11 | 0.59 |  |
|  | EDE-Q total (TP3-2) | 0.30 | 0.16 |  |
|  | BDI (TP3-2) | 0.01 | 0.96 |  |
| Subnetwork connectivity (TP2-1) | BMI (TP2-1) | 0.24 | 0.23 |  |
|  | Age | 0.13 | 0.52 |  |
|  | Age at onset | 0.07 | 0.74 |  |
|  | Illness duration | 0.09 | 0.66 |  |
|  | EDE-Q total (TP2-1) | -0.28 | 0.17 |  |
|  | BDI (TP2-1) | -0.30 | 0.16 |  |
| Subnetwork connectivity (TP3-2) | BMI (TP3-2) | -0.24 | 0.22 |  |
|  | Age | -0.12 | 0.56 |  |
|  | Age at onset | -0.19 | 0.34 |  |
|  | Illness duration | 0.01 | 0.94 |  |
|  | EDE-Q total (TP3-2) | 0.16 | 0.46 |  |
|  | BDI (TP3-2) | 0.04 | 0.88 |  |
| Characteristic path length (TP2-1) | BMI (TP2-1) | 0.00 | 0.99 |  |
|  | Age | -0.07 | 0.71 |  |
|  | Age at onset | -0.16 | 0.44 |  |
|  | Illness duration | 0.02 | 0.94 |  |
|  | EDE-Q total (TP2-1) | -0.01 | 0.96 |  |
|  | BDI (TP2-1) | 0.24 | 0.25 |  |
| Characteristic path length (TP3-2) | BMI (TP3-2) | 0.24 | 0.23 |  |
|  | Age | 0.05 | 0.80 |  |
|  | Age at onset | 0.42 | 0.03 |  |
|  | Illness duration | -0.24 | 0.22 |  |
|  | EDE-Q total (TP3-2) | -0.34 | 0.12 |  |
|  | BDI (TP3-2) | -0.01 | 0.96 |  |
| Clustering coefficient (TP2-1) | BMI (TP2-1) | 0.08 | 0.71 |  |
|  | Age | 0.05 | 0.82 |  |
|  | Age at onset | 0.18 | 0.36 |  |
|  | Illness duration | -0.08 | 0.71 |  |
|  | EDE-Q total (TP2-1) | 0.09 | 0.68 |  |
|  | BDI (TP2-1) | -0.05 | 0.82 |  |
| Clustering coefficient (TP3-2) | BMI (TP3-2) | -0.04 | 0.83 |  |
|  | Age | -0.12 | 0.56 |  |
|  | Age at onset | -0.13 | 0.51 |  |
|  | Illness duration | -0.03 | 0.90 |  |
|  | EDE-Q total (TP3-2) | 0.41 | 0.05 |  |
|  | BDI (TP3-2) | -0.07 | 0.76 |  |
| Modularity (TP2-1) | BMI (TP2-1) | 0.04 | 0.84 |  |
|  | Age | -0.16 | 0.43 |  |
|  | Age at onset | -0.08 | 0.69 |  |
|  | Illness duration | -0.08 | 0.68 |  |
|  | EDE-Q total (TP2-1) | -0.15 | 0.45 |  |
|  | BDI (TP2-1) | 0.07 | 0.75 |  |
| Modularity (TP3-2) | BMI (TP3-2) | 0.29 | 0.14 |  |
|  | Age | 0.04 | 0.82 |  |
|  | **Age at onset** | **0.53** | **0.005*** |  |
|  | Illness duration | -0.33 | 0.10 |  |
|  | EDE-Q total (TP3-2) | -0.09 | 0.69 |  |
|  | BDI (TP3-2) | -0.12 | 0.60 |  |
| *Notes.* Subnetwork connectivity of the subnetwork identified at TP1. BDI = Beck Depression Inventory; EDE-Q = Eating disorder examination questionnaire; TP = time point. * *p* < 0.05 after Holm correction. | | | | |

## Medication effects

Sixteen patients of the AN group were on psychotropic medication at the time of scanning. As this might alter functional brain connectivity [45, 46], we compared mean connectivity within the network identified at TP1 of patients with and without medication. There were no differences between patients receiving medication (*M* = 0.195, *SD* = 0.119) and patients without medication (*M* = 0.149, *SD* = 0.105; *t*(19.86) = 0.13, *p* = .312), suggesting that group differences between AN and HC were not driven by extreme values of medicated patients (Fig. S3).

| 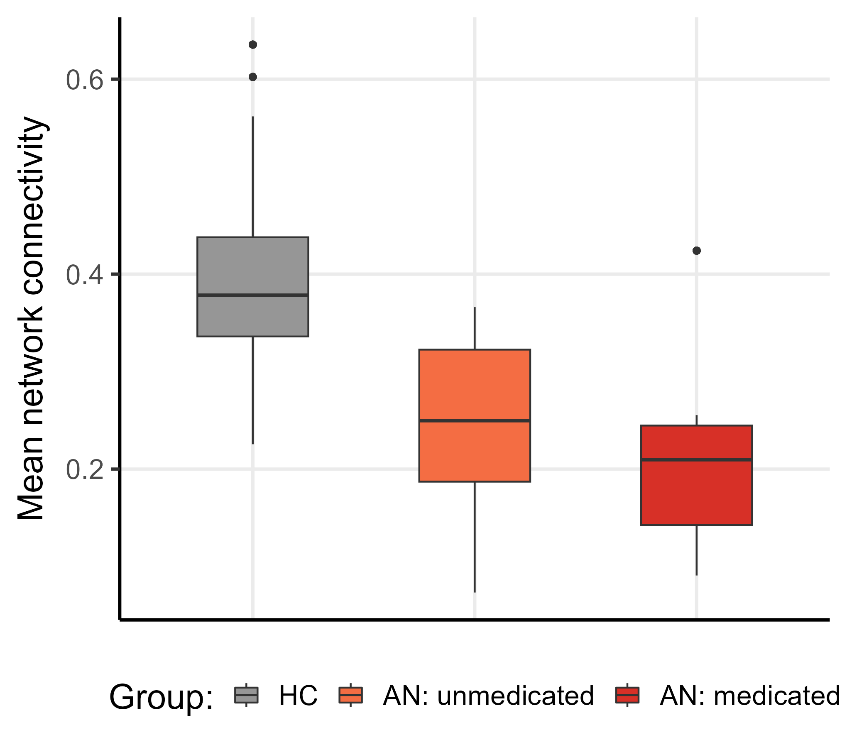 |
| --- |
| **Figure S3.** Boxplots of mean connectivity within the network identified at the first time-point (TP1). The horizontal mark signifies the median per group, edges of the box represent the 25th and 75th percentiles, and the whiskers extend to 1.5 interquartile ranges. AN = Anorexia nervosa group; HC = Healthy control group. |

# References

1. First MB, Spitzer RL, Gibbon M, Williams JBW. Structured Clinical Interview for DSM-IV-TR Axis I Disorders, Research Version, Patient Edition (SCID-I/P). New York, NY: New York State Psychiatric Institute; 2002.

2. American Psychiatric Association. Diagnostic and Statistical Manual of Mental Disorders, Fourth Edition, Text Revision (DSM-IV-TR). Washington, DC: American Psychiatric Association; 2000.

3. Yan C-G, Wang X-D, Zuo X-N, Zang Y-F. DPABI: Data Processing & Analysis for (Resting-State) Brain Imaging. Neuroinformatics. 2016;14:339–351.

4. Jenkinson M, Bannister P, Brady M, Smith S. Improved Optimization for the Robust and Accurate Linear Registration and Motion Correction of Brain Images. NeuroImage. 2002;17:825–841.

5. Friston KJ, Williams S, Howard R, Frackowiak RSJ, Turner R. Movement-Related effects in fMRI time-series. Magn Reson Med. 1996;35:346–355.

6. Yan C-G, Cheung B, Kelly C, Colcombe S, Craddock RC, Di Martino A, et al. A comprehensive assessment of regional variation in the impact of head micromovements on functional connectomics. NeuroImage. 2013;76:183–201.

7. Satterthwaite TD, Elliott MA, Gerraty RT, Ruparel K, Loughead J, Calkins ME, et al. An improved framework for confound regression and filtering for control of motion artifact in the preprocessing of resting-state functional connectivity data. NeuroImage. 2013;64:240–256.

8. Jo HJ, Gotts SJ, Reynolds RC, Bandettini PA, Martin A, Cox RW, et al. Effective Preprocessing Procedures Virtually Eliminate Distance-Dependent Motion Artifacts in Resting State FMRI. J Appl Math. 2013;2013:1–9.

9. Tzourio-Mazoyer N, Landeau B, Papathanassiou D, Crivello F, Etard O, Delcroix N, et al. Automated anatomical labeling of activations in SPM using a macroscopic anatomical parcellation of the MNI MRI single-subject brain. NeuroImage. 2002;15:273–289.

10. Baur V, Hänggi J, Langer N, Jäncke L. Resting-state functional and structural connectivity within an insula-amygdala route specifically index state and trait anxiety. Biol Psychiatry. 2013;73:85–92.

11. Desikan RS, Ségonne F, Fischl B, Quinn BT, Dickerson BC, Blacker D, et al. An automated labeling system for subdividing the human cerebral cortex on MRI scans into gyral based regions of interest. NeuroImage. 2006;31:968–980.

12. Sesack SR, Grace AA. Cortico-Basal Ganglia Reward Network: Microcircuitry. Neuropsychopharmacology. 2010;35:27–47.

13. R Core Team. R: A Language and Environment for Statistical Computing. 2022.

14. Holm S. A Simple Sequentially Rejective Multiple Test Procedure. Scand J Stat. 1979;6:65–70.

15. Robinson D, Hayes A, Couch S. broom: Convert Statistical Objects into Tidy Tibbles. 2021.

16. Wei T, Simko V. corrplot: Visualization of a Correlation Matrix. 2021.

17. Wilke CO. cowplot: Streamlined Plot Theme and Plot Annotations for ggplot2. 2020.

18. Wickham H, François R, Henry L, Müller K. dplyr: A Grammar of Data Manipulation. 2021.

19. Wickham H. forcats: Tools for Working with Categorical Variables (Factors). 2021.

20. Schloerke B, Cook D, Larmarange J, Briatte F, Marbach M, Thoen E, et al. GGally: Extension to ggplot2. 2021.

21. Wickham H, Chang W, Henry L, Pedersen TL, Takahashi K, Wilke C, et al. ggplot2: Create Elegant Data Visualisations Using the Grammar of Graphics. 2021.

22. Pedersen TL. ggraph: An Implementation of Grammar of Graphics for Graphs and Networks. 2021.

23. Auguie B. gridExtra: Miscellaneous Functions for ‘Grid’ Graphics. 2017.

24. Sjoberg DD, Curry M, Larmarange J, Lavery J, Whiting K, Zabor EC. gtsummary: Presentation-Ready Data Summary and Analytic Result Tables. 2021.

25. file. SA. igraph: Network Analysis and Visualization. 2020.

26. Spinu V, Grolemund G, Wickham H. lubridate: Make Dealing with Dates a Little Easier. 2021.

27. Alexander PC. NetworkToolbox: Methods and Measures for Brain, Cognitive, and Psychometric Network Analysis in R. R J. 2018;10:422.

28. Pedersen TL. patchwork: The Composer of Plots. 2020.

29. Revelle W. psych: Procedures for Psychological, Psychometric, and Personality Research. 2021.

30. Henry L, Wickham H. purrr: Functional Programming Tools. 2020.

31. Neuwirth E. RColorBrewer: ColorBrewer Palettes. 2014.

32. Wickham H, Hester J. readr: Read Rectangular Text Data. 2021.

33. Wickham H, Bryan J. readxl: Read Excel Files. 2019.

34. Henry L, Wickham H. rlang: Functions for Base Types and Core R and Tidyverse Features. 2021.

35. Kassambara A. rstatix: Pipe-Friendly Framework for Basic Statistical Tests. 2021.

36. Wickham H. stringr: Simple, Consistent Wrappers for Common String Operations. 2019.

37. Müller K, Wickham H. tibble: Simple Data Frames. 2021.

38. Wickham H. tidyr: Tidy Messy Data. 2021.

39. Wickham H. tidyverse: Easily Install and Load the Tidyverse. 2021.

40. Ehrlich S, Lord AR, Geisler D, Borchardt V, Boehm I, Seidel M, et al. Reduced functional connectivity in the thalamo-insular subnetwork in patients with acute anorexia nervosa. Hum Brain Mapp. 2015;36:1772–1781.

41. Behzadi Y, Restom K, Liau J, Liu TT. A component based noise correction method (CompCor) for BOLD and perfusion based fMRI. NeuroImage. 2007;37:90–101.

42. Muschelli J, Nebel MB, Caffo BS, Barber AD, Pekar JJ, Mostofsky SH. Reduction of motion-related artifacts in resting state fMRI using aCompCor. NeuroImage. 2014;96:22–35.

43. Betzel RF, Byrge L, He Y, Goñi J, Zuo XN, Sporns O. Changes in structural and functional connectivity among resting-state networks across the human lifespan. NeuroImage. 2014;102:345–357.

44. Cao M, Wang JH, Dai ZJ, Cao XY, Jiang LL, Fan FM, et al. Topological organization of the human brain functional connectome across the lifespan. Dev Cogn Neurosci. 2014;7:76–93.

45. McCabe C, Mishor Z. Antidepressant medications reduce subcortical-cortical resting-state functional connectivity in healthy volunteers. NeuroImage. 2011;57:1317–1323.

46. van Wingen GA, Tendolkar I, Urner M, van Marle HJ, Denys D, Verkes R-J, et al. Short-term antidepressant administration reduces default mode and task-positive network connectivity in healthy individuals during rest. NeuroImage. 2014;88:47–53.
